# Association between nurse staffing level in intensive care settings and hospital-acquired pneumonia among surgery patients: result from the Korea National Health Insurance cohort

**DOI:** 10.1017/S0950268824000232

**Published:** 2024-02-08

**Authors:** Yu Shin Park, Il Yun, Suk-Yong Jang, Eun-Cheol Park, Sung-In Jang

**Affiliations:** 1 Department of Public Health, Graduate School, Yonsei University, Seoul, Republic of Korea; 2 Institute of Health Services Research, Yonsei University, Seoul, Republic of Korea; 3 Department of Healthcare Management, Graduate School of Public Health, Yonsei University, Seoul, South Korea; 4 Department of Preventive Medicine, Yonsei University College of Medicine, Seoul, Republic of Korea

**Keywords:** hospital-acquired infection, hospital-acquired pneumonia, nursing staff, quality of care

## Abstract

This study examined the association between the number of nursing staff in intensive care units (ICUs) and hospital-acquired pneumonia (HAP) among surgical patients in South Korea. Data were obtained between 2008 and 2019 from the Korean National Health Insurance Service Cohort Database; 37,706 surgical patients who received critical care services were included in the analysis. Patients with a history of pneumonia 1 year prior to surgery or those who had undergone lung-related surgery were excluded. The ICU nursing management fee is an admission fee that varies based on the grading determined by nurse-to-bed ratio. Using this grading system, we classified four groups from the highest to the lowest level based on the proportion of beds to nurses (high, high-mid, mid-low, and low group). HAP was defined by the International Classification of Disease, 10th revision (ICD-10) code. Multilevel logistic regression was used to investigate the relationship between the level of ICU nurse staffing and pneumonia, controlling for variables at the individual and hospital levels. Lower levels of nurse staffing were associated with a greater incidence of HAP than higher levels of nurse staffing (mid-high, OR: 1.33, 95% CI: 1.12–1.57; mid-low, OR: 1.61, 95% CI: 1.27–2.04; low, OR: 2.13, 95% CI: 1.67–2.71). The intraclass correlation coefficient value was 0.177, and 17.7% of the variability in HAP was accounted for by the hospital. Higher ICU nursing management fee grades (grade 5 and above) in general and hospital settings were significantly associated with an increased risk of HAP compared to grade 1 admissions. Similarly, in tertiary hospitals, grade 2 and higher ICU nursing management fees were significantly associated with an increased risk of HAP compared to grade 1 admissions. Especially, a lower level of nurse staffing was associated with bacterial pneumonia but not pneumonia due to aspiration. In conclusion, this study found an association between the level of ICU nurse staffing and HAP among surgical patients. A lower level of nurse staffing in the ICU was associated with increased rates of HAP among surgical patients. This indicates that having fewer beds assigned to nurses in the ICU setting is a significant factor in preventing HAP, regardless of the size of the hospital.

## Introduction

In the intensive care unit (ICU), advanced medical procedures include the use of invasive monitoring and mechanical devices to support and assist malfunctioning organs or systems, such as the respiratory system [1]. As a result, patients in the ICU are at a higher risk of acquiring hospital-acquired infections (HAIs). Hospital-acquired pneumonia (HAP) is the most prevalent among these infections, representing 26% of all HAI cases [2].

HAP is categorized into two distinct groups: ventilator-associated pneumonia (VAP) and severe pneumonia that occurs while a patient is hospitalized [3]. Specifically, among postoperative patients, pneumonia ranks as the third most frequent complication across all surgical procedures and is linked to higher patient morbidity and mortality rates [4].

It is estimated that about 20% of HAIs can be prevented through improved infection control measures [5]. Numerous international guidelines for the prevention of HAP have been published. Most of these guidelines incorporate strategies that focus on staff education, hand hygiene, patient positioning, ventilator management, the frequency of humidifier changes, and proper suctioning techniques [6–8]. Nurses working in the critical care unit are tasked with various responsibilities related to implementing these infection prevention strategies. Consequently, critically ill patients necessitate increased nursing staff resources and nurses with specialized knowledge and skills to effectively prevent HAP [9].

Several studies have examined the connection between nurse staffing levels and patient health outcomes, including factors like mortality, hospital stay duration, and complications, in both general acute care units and non-critical care units [10–13]. Nonetheless, the nurse staffing requirements for patients with critical care conditions may differ from those in general ward settings. Therefore, studies on nurse staffing that report outcomes in general acute care settings may not be applicable to the ICU, and there is a shortage of studies regarding nurse staffing levels, specifically in intensive care settings in Korea.

Furthermore, determining what constitutes satisfactory nurse staffing levels remains a topic of debate in hospitals around the world [9]. In South Korea, a financial incentive program was introduced in 1999. It provides higher inpatient nursing fees to hospitals that establish nursing staff standards using a ratio of beds to registered nurses (RNs). The grading of nursing levels is determined by the type of hospital, differentiating between general and tertiary hospitals. This system was extended to the ICU setting starting in 2008 [14]. Annually, the government collects information on the total number of nursing staff and the number of hospital beds in almost all hospitals and provides differentiated payment based on these data.

Following this policy, it is possible to clearly confirm the ICU nurse staffing level in all hospitals using the claim code. It is hence possible to evaluate the association between nurse staffing level and HAP nationwide. Therefore, this study examined the association between nurse staffing level and HAP in operative patients in the ICU using nationwide representative cohort data. This might enrich international evidence of the impact of nursing staff in different national and organizational contexts.

## Method

### Study design and data source

This study was a cross-sectional analysis of claim data from the Korean National Health Insurance (NHI) Service National Sample Cohort from 2008 to 2019. The NHI data (NHID) consist of medical claim records that represent nationally representative random samples of the Korean population, accounting for approximately 2.2% of the entire population. The Korean National Health Insurance Service (NHIS) supplies data for academic research and policy formulation, encompassing all claims data collected through the NHIS application procedure. Patients in the cohort were monitored, except in cases of exclusion due to death or relocation. The National Health Insurance Database (NHID) contains details regarding socioeconomic status and clinically ascertained International Classification of Disease, 10th revision (ICD-10) codes [15]. The present study was approved as exempt by the Institutional Review Board of Yonsei University’s Health System, and informed consent was waived by the Ethics Committee as NHID pseudonymized patient data for research purposes (IRB number: 4-2023-1190).

### Grade of the nursing management fee

In South Korea, a fee-for-service system operates under payment system. The number of beds in hospitals varies by hospital type, but under the Medical Service Act, they can be roughly categorized into about four types: tertiary hospitals, general hospitals, hospitals, and clinics. Typically, clinics primarily handle outpatient cases and hospitals have 30 or more beds, while general hospitals have 100 or more beds and offer services in at least nine different medical specialties. Tertiary general hospitals, on the other hand, are defined by their specialization in providing highly advanced and specialized medical care for severe or complex conditions and offer services in more than 20 medical specialties.

Since 2008, the inpatient fees for ICUs have been determined based on the “Grade of the Nursing Management Fee,” which is linked to the level of nursing staff availability. Tertiary hospitals are categorized into grades ranging from 1 to 5, and general hospitals and hospitals are categorized into grades ranging from 1 to 9 based on the ratio of the number of beds to the number of nurses. This system was implemented to address the undesirable situation where the quality of nursing care in ICUs was compromised due to inadequate nursing staffing levels at hospitals. The grades are as follows, as shown in Supplementary Table 1. These grades were identified by claim codes.

### Participants

We extracted patients who were admitted to critical care units in general hospitals, hospitals, or tertiary hospitals by identifying them by the claim fee code (*n* = 84,397). We excluded patients (1) who had received lung surgery, according to the surgery claim fee code (*n* = 1,226); (2) who did not undergo surgery during their hospitalization, and we selected those who had checked “No” for “Underwent surgery during admission” (*n* = 40,673); (3) who were diagnosed with any type of pneumonia in the year before admission to the hospital (*n* = 350); and (4) who admitted to NICU or PICU (*n* = 2,301); and (5) with missing covariate values (*n* = 3,247). The total number of study participants in the 2008–2019 period was 36,600.

### Level of nursing staffing

To divide tertiary hospitals, general hospitals, and hospitals into quartiles based on the level of nursing staffing, we categorized grades 1–2 for tertiary hospitals, general hospitals, and hospitals as the high group, grades 3–4 as the mid-high group, grades 5 for tertiary hospitals, grades 5–6 for general hospitals and hospitals as the mid-low group, and grades 7–9 for general hospitals as the low group. We conducted a sensitivity analysis by dividing the type of hospitals (tertiary, general hospitals, and hospitals) and each nursing grade to further investigate the precise associations with HAP.

### Hospital-acquired pneumonia

Our dependent variable was HAP, which was defined using data obtained from all hospitalizations with diagnoses specified by ICD-10 codes J13, J14, J150-J159, J168, J180, J188, J690, and J698. To minimize the inclusion of patients with a history of pneumonia, we excluded individuals with a pulmonary disease history within 1 year before surgery. Additionally, those who were admitted for pulmonary surgery were also excluded from the study population. Furthermore, the fact that patients with pneumonia are not recommended for surgery might increase the accuracy of identifying HAP. Furthermore, we defined our second dependent variable by categorizing HAP into two types: bacterial pneumonia (J15) and aspiration pneumonia (J69).

### Variables

Individual-level variables included various categories, such as sociodemographic factors, socioeconomic status, health status, and treatment. Sociodemographic aspects covered age as a continuous variable, gender (male or female), and residential area (metropolitan, urban, or rural). Socioeconomic status was represented by household income (categorized as high, middle, or low). Health status encompassed disability status (yes or no) and the Charlson comorbidity index with three categories (1, 2, or ≥3). Medical utilization considered whether the patient received invasive mechanical ventilation (yes or no) and renal replacement therapy (yes or no) and total days in the ICU divided into tertiles: The group of individuals who were hospitalized in the ICU for just 1 day was categorized as the low group, those who stayed in the ICU for 2 to 4 days were classified as the middle group, and those who were in the ICU for 5 days or more were designated as the high group.

Hospital-level variables were based on hospital characteristics, namely the type of hospital (tertiary hospital, general hospital, or hospital), location of hospital (metropolitan, city, or rural location), and the number of doctors per bed which was calculated for each hospital by dividing the total number of beds by the total number of doctors and then categorizing them into quartiles. Hospitals with a value equal to or greater than 0.54 were labelled as the high group, those with values exceeding 0.41 but less than 0.54 were classified as the high-mid group, those with values higher than 0.23 but less than 0.41 were considered the mid-low group, and hospitals with values equal to or less than 0.23 were designated as the low group.

### Statistical analysis

The chi-square test and *t*-test were used to determine significant differences in variables between participants who had and did not have a diagnosis of HAP. The null hypothesis (H0) was that there was no difference between groups for a particular variable. To investigate the effect of individual- and hospital-level variables on an individual’s likelihood of HAP, we used two-level hierarchical models that assessed the relationship between HAP and both individual- and hospital-level variables.

To investigate the relationships between individual factors at level 1 and hospital-related factors at level 2 with our study outcomes, we employed generalized linear mixed models for our multilevel regression analysis. We constructed four models for this analysis. The initial model served as a null model with no variables. In the second model (model 2), we introduced individual-level variables to assess their impact on HAP. Model 3 focused on the influence of hospital-level variables, with the addition of 200 hospitals as random effects to explore their unique contributions. Finally, our fourth model (model 4) incorporated both individual and hospital-level variables. We utilized intraclass correlations (ICCs) to evaluate whether there was significant variation between different groups compared to variation within those groups. The ICC is calculated as the ratio of the variance between clusters to the total variance. Additionally, we conducted subgroup analyses to examine the associations between the level of nursing staffing and HAP, stratified by the severity of the patient’s illness (including factors such as invasive mechanical ventilation, ICU stay, and primary admission diagnosis) as well as the hospital’s characteristics (hospital location). We used PROC GLIMMIX to estimate a generalized linear mixed model with a binary distribution and logit link function. Odds ratios (ORs) and 95% confidence intervals (CIs) were also calculated. Data were analyzed using SAS (version 9.4; SAS Institute Inc., Cary, NC, USA).

## Results

Table 1 presents the general characteristics of the study participants. Of the 36,660 participants included in our study, 2,231 (6.1%) developed HAP. Among those who received ventilator care, 1,364 patients (12.1%) experienced HAP, while only 3.4% of those who did not receive ventilator care had HAP. Furthermore, in the group with an ICU stay of 5 days or more, 15.1% experienced HAP. Among patients who underwent surgery at the low level of nursing staff, 10.7% had HAP, and patients with HAP were higher in tertiary general hospitals (3.8%) but lower than in general hospitals (7.8%). Among patients who underwent surgery at hospitals in rural areas, 7.9% had HAP.

**Table 1.** General characteristics of the study population

Variables	Hospital-acquired pneumonia						
	Total		Yes		No		*p*-value
	*n*	%^a^	*n*	*%* ^b^ (SD)	*n*	*%* ^b^	
	36,660	100.0	2,231	6.1	34,429	93.9	
Individual level							
Sex							0.0002
Men	21,525	58.7	1,393	6.5	20,132	93.5	
Women	15,135	41.3	838	5.5	14,297	94.5	
Age (Mean/SD)							<.0001
Region							0.0019
Metropolitan	7,186	19.6	399	5.6	6,787	94.4	
City	16,634	45.4	976	5.9	15,658	94.1	
Rural	12,840	35.0	856	6.7	11,984	93.3	
Household income							0.0043
Low	7,156	19.5	453	6.3	6,703	93.7	
Middle	14,053	38.3	754	5.4	13,299	94.6	
High	15,421	42.1	994	6.4	14,427	93.6	
Invasive mechanical ventilator							<.0001
Yes	11,312	30.9	1,364	12.1	9,948	87.9	
No	25,348	69.1	867	3.4	24,481	96.6	
Renal replacement therapy							0.0036
Yes	2,382	6.5	241	10.1	2,141	89.9	
No	34,278	93.5	1990	5.8	32,288	94.2	
Charlson comorbidity index							0.6270
0–1	3,032	8.3	173	5.7	2,859	94.3	
2	2,725	7.4	163	6.0	2,562	94.0	
3≤	30,903	84.3	1895	6.1	29,008	93.9	
Total days in ICU							<.0001
Tertile 1	14,168	38.6	189	1.3	13,979	98.7	
Tertile 2	11,782	32.1	427	3.6	11,355	96.4	
Tertile 3	10,710	29.2	1,615	15.1	9,095	84.9	
Diagnosis for adimission							<.0001
Oncological	7,393	20.2	237	3.2	7,156	96.8	
Neurological	1821	5.0	205	11.3	1,616	88.7	
Gastrointestinal	4,700	12.8	176	3.7	4,524	96.3	
Cardiovascular disease	12,024	32.8	979	8.1	11,045	91.9	
Other	10,722	29.2	634	5.9	10,088	94.1	
Hospital level							
Level of nursing staff							<.0001
High	20,348	55.5	837	4.1	19,511	95.9	
Mid–high	8,653	23.6	630	7.3	8,023	92.7	
Mid–low	4,073	11.1	379	9.3	3,694	90.7	
Low	3,586	9.8	385	10.7	3,201	89.3	
Type of hospital							<.0001
Tertiary hospital	15,754	43.0	605	3.8	15,149	96.2	
General hospital and hospital	20,906	57.0	1,626	7.8	19,280	92.2	
Location of hospital							<.0001
Metropolitan	12,166	33.2	506	4.2	11,660	95.8	
City	16,174	44.1	1,070	6.6	15,104	93.4	
Rural	8,320	22.7	655	7.9	7,665	92.1	
Bed to doctor ratio							<.0001
High	9,533	26.0	301.0	3.2	9,232	96.8	
Mid–high	8,739	23.8	497.0	5.7	8,242	94.3	
Mid–low	9,511	25.9	595.0	6.3	8,916	93.7	
Low	8,422	23.0	383.0	4.5	8,039	95.5	

*p* values reflect *t*-tests for continuous variables and *χ*^2^ tests for dichotomous/categorical variables.

a Column percentage.

b Row percentage.

Table 2 presents the findings of the multilevel logistic regression analysis of the association between ICU nursing staff and HAP among surgical patients. Between-area variance in HAP was 0.708 (standard error: 0.085) in model 1, and the intraclass correlation coefficient (ICC) value was 0.177, indicating that 17.7% of the variability in HAP was accounted for by the hospital. In model 2, after including individual-level variables, the percentage change in variation was found to be 8.9%. Patients who received invasive ventilator care were at increased risk of HAP than those who did not (OR: 2.56, 95% CI 2.30–2.85), and patients who were hospitalized longer in ICU were also at increased risk of HAP (OR: 7.05; 95% CI: 5.98–2.63). In model 3, low levels of nurse staffing were associated with a higher incidence of HAP (low group: OR:1.88; 95% CI: 1.49–2.37). Model 4 included individual and hospital variables. Adjustment for both individual level and hospital level (model 4) led to a further reduction in area-level variance. Model 4 also presented that low levels of nurse staffing were associated with a higher incidence of HAP (mid-high, OR: 1.33, 95% CI: 1.12–1.57; mid-low, OR: 1.61, 95% CI: 1.27–2.04; low, OR: 2.13, 95% CI: 1.67–2.71).

**Table 2.** Adjusted odds ratio of HAP by characteristics of individual- and hospital-level, multi-level model

Variables	Hospital-acquired pneumonia														
	Model 1			Model 2				Model 3				model 4			
	OR	95% CI		OR	95% CI			OR	95% CI			OR	95% CI		
Individual level(fixed effect)															
Sex															
Men				1.00								1.00			
Women				0.73	(0.66–0.80)							0.73	0.66–0.80		
Age (years)				1.02	(1.02–1.03)							1.02	1.02–1.03		
Region															
Metropolitan				1.00								1.00			
City				0.99	(0.84–1.17)							0.93	0.78–1.11		
Rural				1.02	(0.86–1.23)							0.93	0.77–1.13		
Household income															
Low				1.00								1.00			
Middle				1.01	(0.89–1.15)							1.00	0.88–1.14		
High				1.11	(0.97–1.25)							1.09	0.96–1.24		
Invasive mechanical ventilator															
Yes				2.56	(2.30–2.85)							2.66	2.39–2.96		
No				1.00								1.00			
Renal replacement therapy															
Yes				0.98	(0.83–1.14)							1.01	0.86–1.18		
No				1.00								1.00			
Charlson comorbidity index															
0–1				1.00								1.00			
2				0.95	(0.75–1.22)							0.98	0.77–1.25		
≤3				0.95	(0.79–1.15)							0.99	0.82–1.19		
Total days in ICU															
Tertile 1				1.00								1.00			
Tertile 2				2.20	(1.84–1.84)							2.20	1.84–2.63		
Tertile 3				7.05	(5.98–2.63)							6.93	5.88–8.17		
Diagnosis for admission															
Oncological				1.00								1.00			
Neurological				2.01	(1.62–2.50)							2.00	1.61–2.49		
Gastrointestinal				0.79	(0.64–0.98)							0.78	0.63–0.97		
Cardiovascular disease				1.50	(1.28–1.76)							1.49	1.27–1.74		
Other				1.28	(1.08–1.52)							1.28	1.08–1.51		
*Hospital level*															
Level of nursing staff															
High								1.00				1.00			
Mid–high								1.21	(1.03–1.41)			1.33	1.12–1.57		
Mid–low								1.50	(1.20–1.88)			1.61	1.27–2.04		
Low								1.88	(1.49–2.37)			2.13	1.67–2.71		
Type of hospital															
Tertiary hospital								1.00				1.00			
General hospital and hospital								1.24	(0.97–1.59)			1.23	0.96–1.58		
Location of hospital															
Metropolitan								1.00				1.00			
City								1.01	(0.76–1.34)			1.12	0.83–1.50		
Rural								1.06	(0.78–1.45)			1.09	0.79–1.52		
Number of physician per bed															
High								1.00				1.00			
Mid–high								1.15	(0.94–1.40)			1.12	(0.91–1.38)		
Mid–low								1.16	(0.89–1.51)			1.14	(0.86–1.50)		
Low								1.28	(0.93–1.75)			1.29	0.93–1.78		
Between area variance (SE)	0.708 (0.085)*			0.645 (0.082)*				0.519 (0.068)*				0.444 (0.0618)*			
Percentage change in variation		–		8.9%				26.7%				37.3%			
Model Fitness															
−2 Log Likelihood	15,262.83			12,866.58				15,267.74				13,367.03			
Intraclass correlation coefficient (%)^a^	17.7%														

OR, odds ratio; CI, confidence interval; SE, standard error.

a 17.7% of the variability in HAP is accounted for by the hospital in the study.

* *p* < 0.0001.

Table 3 shows the association between ICU nurse staffing and HAP by covariate. Among patients who received VT care, patients in hospitals with a low level of nurse staffing had a significantly higher likelihood of HAP (mid-high, OR: 1.34, 95% CI: 1.09–1.64; low, OR: 1.82, 95% CI: 1.31–2.51). Patients whose length of stay in the ICU fell in tertile 2 had a greater association with HAP (mid-high, OR: 1.67, 95% CI: 1.20–2.32; mid-low, OR: 2.31, 95% CI: 1.48–3.62; low, OR: 2.70, 95% CI: 1.69–4.29). Among patients who utilized hospitals in the city, the lower the level of nurse staffing, the higher the association with HAP (mid-high, OR: 1.38, 95% CI: 1.10–1.74; mid-low, OR: 1.80, 95% CI: 1.27–2.56; low, OR: 2.39, 95% CI: 1.70–3.36).

**Table 3.** Subgroup analysis stratified by covariates

Variables	Hospital-acquired pneumonia												
	Level of nursing staff												
	High	Mid-high				Mid-low				Low			
	OR	OR	95% CI			OR	95% CI			OR	95% CI		
Invasive mechanical ventilator													
Yes	1.00	1.34	(1.09–1.64)			1.46	(1.07–2.00)			1.82	(1.31–2.51)		
No	1.00	1.46	(1.10–1.92)			2.01	(1.42–2.84)			2.74	(1.93–3.89)		
Total days in ICU													
Low	1.00	2.06	(1.12 3.49)			2.85	(1.38–5.91)			3.23	(1.52–6.86)		
Middle	1.00	1.67	(1.20–2.32)			2.31	(1.48–3.62)			2.70	(1.69–4.29)		
High	1.00	1.18	(0.97–1.45)			1.37	(1.03–1.81)			1.37	(1.03–1.81)		
Diagnosis for admission													
Oncological	1.00	1.53	(1.00–2.33)			1.69	(0.83–3.46)			2.41	(1.21–4.80)		
Neurological	1.00	1.11	(0.63–1.96)			2.57	(1.18–5.62)			2.43	(1.09–5.42)		
Gastrointestinal	1.00	1.74	(0.99–3.04)			1.61	(0.76–3.40)			2.01	(0.95–4.27)		
Cardiovascular disease	1.00	1.40	(1.09–1.81)			1.68	(1.20–2.37)			2.50	(1.76–3.56)		
Other	1.00	1.23	(0.90–1.66)			1.48	(0.99–2.24)			1.62	(1.05–2.49)		
Hospital level													
Location of hospital													
Metropolitan	1.00	1.60	(1.16–2.19)			2.97	(1.61–5.47)			1.61	(0.88–2.95)		
City	1.00	1.38	(1.10–1.74)			1.80	(1.27–2.56)			2.39	(1.70–3.36)		
Rural	1.00	1.07	(0.75–1.80)			1.16	(0.75–1.80)			1.74	(1.07–2.81)		

Adjusted for all covariates.

Table 4 presents a subgroup analysis that assessed the relationship between grade of the nursing management fee in ICU and HAP by hospital type. The likelihood of HAP was higher when patients were admitted to ICUs with higher nursing management fee grades, specifically grade 5 and above, compared to grade 1 admissions, both in general hospital and hospital settings. Likewise, within tertiary hospitals, ICU admissions with nursing management fees of grade 2 or higher were also significantly linked to an increased association of HAP when compared to grade 1 admissions.

**Table 4.** Association between grade of the nursing management fee in ICU and HAP by type of hospital

Variables	Hospital-acquired pneumonia			
	OR	95% CI		
*Criteria of grade of the nursing management fee in ICU*				
Total hospital				
1 grade	1.00			
2 grade	1.39	(1.11–1.75)		
3 grade	1.83	(1.42–2.35)		
4 grade	1.83	(1.33–2.51)		
5 grade	2.27	(1.64–3.16)		
6 grade	2.25	(1.61–3.15)		
7 grade	3.08	(2.24–4.24)		
8 grade	2.57	(1.71–3.85)		
9 grade	3.13	(2.02–4.85)		
Tertiary hospital				
1 grade	1.00			
2 grade	1.45	(1.10–1.92)		
3 grade	1.98	(1.35–2.92)		
4 grade	3.52	(0.79–15.64)		
5 grade	1.76	(0.79–3.90)		
General hospital and hospital				
1 grade	1.00			
2 grade	1.09	(0.74–1.60)		
3 grade	1.39	(0.94–2.05)		
4 grade	1.38	(0.90–2.13)		
5 grade	1.75	(1.12–2.74)		
6 grade	1.71	(1.10–2.66)		
7 grade	2.35	(1.53–3.61)		
8 grade	2.01	(1.22–3.30)		
9 grade	2.35	(1.39–3.97)		

Adjusted for all covariates.

Table 5 shows the relationship between the level of nurse staffing and the type of HAP. A lower level of nurse staffing had a stronger association with bacterial pneumonia (mid-high, OR: 1.88, 95% CI: 1.41–2.51; mid-low, OR: 2.44, 95% CI: 1.68–3.54; low: OR: 3.32, 95% CI: 2.28–4.85). Also, as the nursing staff level decreased, the association with aspiration pneumonia increased, although it was not statistically significant (mid-high, OR: 1.07, 95% CI: 0.87–1.32; mid-low, OR: 1.16, 95% CI: 0.84–1.58; low: OR: 1.25, 95% CI: 0.90–1.74).

**Table 5.** Association between level of nursing staff and type of pneumonia

Variables	Hospital–acquired pneumonia							
	Bacterial pneumonia				Pneumonia due to aspiration			
	OR	95% CI			OR	95% CI		
Level of nursing staff								
High	1.00				1.00			
Mid–high	1.88	1.41–2.51			1.07	0.87–1.32		
Mid–low	2.44	1.68–3.54			1.16	0.84–1.59		
Low	3.32	2.28–4.85			1.25	0.90–1.74		

Adjusted for all covariates.

## Discussion

Our study investigated the association between the level of nurse staffing in the ICU and HAP among surgical patients using National Health Insurance cohort data. Our results showed that surgical patients experiencing lower levels of nurse staffing in the ICU were more likely to develop HAP than those experiencing standard levels of nurse staffing. We determined factors associated with HAP at both individual and hospital levels. Also, our results might suggest the association with HAP varies across hospitals and that differences in hospital conditions should be considered to prevent HAI.

National representative data on the relationship between nurse staffing levels and HAP are scarce in previous studies. Some reports on the relationship between staffing level and HAP are available [9]. A systematic review investigated the relationship between healthcare-associated pneumonia and nurse staffing levels. Almost all studies found a relationship between the proportion of total hours of nursing care provided by RNs or the number of registered hours per day and pneumonia among patients [9, 11, 16–18]. However, there have been few studies on how many RNs per patient are appropriate to improve the quality of care. Additionally, some studies found no link between staffing levels and the risk of pneumonia. However, this may be due to the severity of patients’ conditions, hospital size, and hospital-specific differences. Our study design reflected both hospital and individual health characteristics, using a multilevel analysis.

Previous studies demonstrated that adequate nurse staffing levels are linked to positive patient outcomes in the ICU [19, 20]. Patients admitted to the ICU in general hospital with a grade 5 of ICU nursing management fee showed a significantly increased association with HAP compared to patients admitted to a grade 1. Patients admitted to ICU in tertiary hospital with a grade 2 of ICU nursing management fee showed a significantly increased association with HAP compared to patients admitted to a grade 1. This suggests that in the ICU setting, the number of beds assigned to nurses is a meaningful factor in preventing HAP in hospitals of all sizes. Another study in Korea showed that less than grade 5 of ICU nursing management fee in the ICU was associated with a shorter length of stay [21]. The results showed that a similar grade of the nursing management fee was associated with acceptable quality of medical care.

Furthermore, we found a relationship between the level of nurse staffing and pneumonia; in particular, staffing differences were associated with bacterial pneumonia rather than aspiration pneumonia. Various studies have shown that risk factors for aspiration pneumonia are related to individual health conditions, not hospital conditions, such as oral health, alcohol consumption, dehydration, and dementia, and studies related to hospital characteristics and nurse staffing levels have been limited [22–25]. However, patients, staff, and equipment clearly contribute to bacterial transmission, increasing the risk of infection [26, 27]. A low level of nurse staffing might result in nurses caring for more patients, which can lead to increased workload, which is associated with HAP [11].

Our results showed that the relationship between nurse staffing levels and HAP differs according to according to patients’ medical needs or hospital location. The association between nurse staffing level and HAP appeared to differ according to patients’ diagnoses. There was a relatively low association between nursing staff levels and HAP for hospitals located in rural areas. Due to the characteristics of the healthcare delivery system in Korea, patients with severe and acute conditions frequently utilize hospitals in the metropolitan or city, and most such hospitals have a high level of nurse staffing.

This study had several limitations. First, we only considered ICUs used by adults, excluding neonatal or paediatric ICUs. This did not permit derivation of the association between pneumonia and the overall level of intensive care nurse staffing in Korea. Further research is needed because there is a large difference in health characteristics when analyzing the nursing level for each population. Second, since there was a claim data limitation regarding clearly distinguishing HAP, we screened for HAP by specific clerical definition. However, we made efforts to improve the accuracy of HAP by excluding individuals with a history of pneumonia within the past year and those who had undergone lung-related surgery before surgery. Third, after 2019, a financial incentive system related to the level of nurse staffing changed the method of calculation from nurse-to-bed ratio to nurse-to-patient ratio. Our data used the nurse-to-bed ratio in the study period before the calculation method changed. Therefore, additional research on the appropriate number of nurses per patient is needed using the data after the change. Fourth, our data did not consider the characteristics of nursing staff, such as the nurses’ educational level and clinical experience. However, the grade of nurse staffing is highly accurate because it is measured as the number of nurses reported to the government every year compared with the number of ICU beds in Korean medical institutions.

## Conclusion

In conclusion, this study found an association between the level of nurse staffing and HAP in surgical patients. A lower level of nurse staffing in the ICU had a strong relationship with HAP among surgical patients. This indicates that having fewer beds assigned to nurses in the ICU setting is a significant factor in preventing HAP regardless of the size of the hospital.

## Supporting information

Park et al. supplementary material Park et al. supplementary material

## Data Availability

The data that support the findings of this study are available from the corresponding author, SI Jang, upon reasonable request.

## Supplementary material

For supplementary material accompanying this paper visit http://doi.org/10.1017/S0950268824000232. click here to view supplementary material
