# Supplementary material for: Association between nurse staffing level in intensive care settings and hospital-acquired pneumonia among surgery patients: result from the Korea National Health Insurance cohort
Source: Epidemiol Infect. 2024 Feb 8;152:e62. doi: 10.1017/S0950268824000232 (PMC11062778; doi:10.1017/S0950268824000232)
Supplement: Park et al. supplementary material [file S0950268824000232sup001.docx]

| **Supplementary table 1. Criteria of Grade of the ICU Nursing Management Fee** | | | |
| --- | --- | --- | --- |
| **Criteria of Grade of the Nursing Management Fee** | | | |
| **Tertiary hospital** | | **General hospital & Hospitals** | |
| **Grade of the ICU Nursing Management Fee** | **The ratio of the number of beds to the number of nurses** | **Grade of the ICU Nursing Management Fee** | **The ratio of the number of beds to the number of nurses** |
| 1(better) | ~0.5:1 | 1(better) | ~0.5:1 |
| 2 | 0.5:1~0.63:1 | 2 | 0.5:1~0.63:1 |
| 3 | 0.63:1~0.77:1 | 3 | 0.63:1~0.77:1 |
| 4 | 0.77:1~0.88:1 | 4 | 0.77:1~0.88:1 |
| 5(worse) | 0.88:1~ | 5 | 0.88:1~1:1 |
|  |  | 6 | 1:1~1.25:1 |
|  |  | 7 | 1.25:1~1.5:1 |
|  |  | 8 | 1.5:1~2.0:1 |
|  |  | 9(worse) | 2.0:1~ |
